# Increasing Intelligence in Inter-Vehicle Communications to Reduce Traffic Congestions: Experiments in Urban and Highway Environments

**DOI:** 10.1371/journal.pone.0159110

**Published:** 2016-08-15

**Authors:** Rodolfo I. Meneguette, Geraldo P. R. Filho, Daniel L. Guidoni, Gustavo Pessin, Leandro A. Villas, Jó Ueyama

**Affiliations:** 1 University of Ottawa, Ottawa, Ontario, Canada; 2 Federal Institute of São Paulo (IFSP), Catanduva, São Paulo, Brazil; 3 Institute of Mathematics and Computer Sciences (ICMC), University of São Paulo (USP), São Carlos, Brazil; 4 Computer Science Department, Federal University of Sao Joao del-Rei, Sao Joao del-Rei, Brazil; 5 Applied Computing Lab, Vale Institute of Technology, Belem, Para, Brazil; 6 Institute of Computing (IC), University of Campinas (UNICAMP), Campinas, Sao Paulo, Brazil; 7 Ming Hsieh Department of Electrical Engineering, University of Southern California, Los Angeles, United States of America; Chongqing University, CHINA

## Abstract

Intelligent Transportation Systems (ITS) rely on Inter-Vehicle Communication (IVC) to streamline the operation of vehicles by managing vehicle traffic, assisting drivers with safety and sharing information, as well as providing appropriate services for passengers. Traffic congestion is an urban mobility problem, which causes stress to drivers and economic losses. In this context, this work proposes a solution for the detection, dissemination and control of congested roads based on inter-vehicle communication, called INCIDEnT. The main goal of the proposed solution is to reduce the average trip time, CO emissions and fuel consumption by allowing motorists to avoid congested roads. The simulation results show that our proposed solution leads to short delays and a low overhead. Moreover, it is efficient with regard to the coverage of the event and the distance to which the information can be propagated. The findings of the investigation show that the proposed solution leads to (i) high hit rate in the classification of the level of congestion, (ii) a reduction in average trip time, (iii) a reduction in fuel consumption, and (iv) reduced CO emissions

## 1 Introduction

In the last decade, the use of mobile devices, ad hoc communication and ubiquitous computing have changed people’s lives, by allowing the exchange of information anywhere and anytime. It is expected that the use of mobile communications in vehicles will be a reality in a few years’time, since the automobile industry, governments and universities around the world are devoting resources to creating a safe transportation system [1–4]. Vehicular Ad Hoc Networks (VANETs) or simply ‘vehicular networks’, are a special type of mobile ad hoc networks, where vehicles are equipped with wireless communication and processing capabilities. Such vehicles can create a mobile network as they move along roads [1, 5].

The establishment of a vehicle network will be a major step forward towards building intelligent transportation systems. A growing number of manufacturers are equipping vehicles with an on-board computer, wireless communication devices, sensors and navigation systems (e.g. GPS) and in this way creating large-scale vehicular networks [5–7]. By using different kinds of sensor devices (e.g. to detect road and weather conditions, the state and situation of vehicles etc.), cameras, computers and communication services, vehicles are able to collect, process, interpret and send information that assists the drivers.

Vehicular networking applications can be clustered into three categories [2]: security, entertainment, and driver assistance. Security- related applications in traffic are intended to provide information to the driver about dangerous road conditions, such as weather, traffic jams, accidents, etc. The purpose of these applications is to propagate emergency information so that the driver can react to these situations in a timely manner. The entertainment applications support the Internet access, advertising, content sharing, chats and other services. The driver support applications involve the exchange of information that can help the driver, such as the location of gas stations, restaurants, and toll roads. All these applications comprise the so-called intelligent transportation system (ITS) [2, 8]. The main goal of the ITS is to provide the driver and passengers with a safe, pleasant and efficient means of transport. Efficient solutions are related to the increase of maneuverability and the reduction of traffic and pollution to make the likely travel time more predictable and improve the management and control of the road network.

Some of the described applications use data dissemination protocols to carry out a message exchange between vehicles [9, 10]. The designing of data dissemination protocols plays an important role since the topology of a vehicular network may change dramatically over time. During the rush hour, there is a large number of vehicles on the same road. On the other hand, late at night, there are just a few vehicles using the same road. Thus, the design of data dissemination protocols is challenging and is one of the most important tasks in vehicular ad-hoc networks. The protocol designer must take account of the inherent features of VANETs, in which the vehicles’ topology is highly dynamic and disconnections are no exception.

Data dissemination performed by vehicular network applications usually involves broadcasting messages [11, 12]. When a vehicle sends a broadcast packet, all the neighbor vehicles can receive the message and forward it. However, data dissemination protocols based on broadcast/flooding-based messages can give rise to two problems: (i) broadcast storm and (ii) intermittent network. The broadcast storm problem arises when multiple vehicles send/forward messages at the same time. During traffic jams, the density of vehicles in the same region, transmitting messages through broadcasts, may lead to colliding messages and network congestion causing a further delay in the medium access (MAC) layer. The network partition problem is basically caused by the density of vehicles in the network or a region of it [13]. In a sparse network, a vehicle might not have any vehicle within its communication range and the routing process may not be successfully performed. However, if the data dissemination protocol is aware of network disruption, the protocol can change its operation to a store-carry-forward scheme and thus forward the message whenever possible through the vehicles. Although there are several solutions in the literature related to data dissemination protocols, there are few protocols that combine both problems in order to design an efficient data dissemination protocol that is able to overcome both problems together.

In specific terms, data dissemination protocols that are designed to detect and suggest different routes to reduce congestion in urban scenarios must take into account the travel time and network density so that congestions can be predicted [14]. After detecting a traffic jam, the protocol creates different routes to avoid the congestion. However, the protocol must take account of both the broadcast storm and intermittent network problems when disseminating this information to the nearby vehicles. The steps taken to avoid traffic jams may undergo several communication problems, such as congestion estimation levels with low accuracy, reliability issues, redundant data propagation and flooding- based issues.

In this context, this work proposes INCIDEnT, an INtelligent protocol of CongestIon DETection for urban and highway environments. INCIDEnT is based on a neural network which is able to (i) detect the different level of congestion; (ii) classify the congestion levels and (iii) suggest new routes to avoid the congestion. The proposed protocol was designed to reduce traffic jams and prevent new vehicles from reaching them, improve the vehicle flow, reduce the travel time, as well as to reduce CO emissions and fuel consumption. INCIDEnT was evaluated by means of different metrics, such as: (1) network density; (2) dissemination coverage; (3) communication delay; (4) transmitted packets; (5) CO emission; (6) fuel consumption and (7) trip time.

The rest of this work is structured as follows. In the next section, there is an overview of the main existing approaches for vehicle congestion detection and data dissemination in VANETs. The proposed solution is described in Section 3, while a detailed performance evaluation and the results of the simulation are shown in Section 4. Finally, Section 5 summarizes the conclusion and make suggestions for future work.

## 2 Related Work

This section will describe some studies that have investigated the question of data dissemination and the way it can be designed to propagate particular information and protocols that involve on ITS to reduce congestion on the roads.

### 2.1 Data Dissemination

The simplest way to perform data dissemination in a communication network is by flooding. Despite its simplicity, this approach can causes a broadcast storm problem when the network density increases and does not perform well in sparse networks, since it is unable to handle intermittent network. In view of this, current techniques for data dissemination mainly focus on the way packets are forwarded in a way that enables them to handle both the intermittent network or the broadcast storm problem. Several proposals can be found: position-based, statistically-based, distance-based, (local) topology-based, timer-based, and map-based, as discussed in the following [15–19].

Linger [16] is a protocol used to transmit information inside a RoI. To this end, Linger proposes using an index that is computed locally by vehicles and is based on the distance to the center point of the RoI, the angle relative to the RoI center point, and the speed of the vehicles. This protocol is an entirely distributed solution in which vehicles compute a comparable index that indicates how suitable they seem to be as replicas. The index takes into account the vehicle’s speed, direction, and distance to the RoI.

Kim et al. [17] propose the Distance Based Relay Selection (DBRS), which is a simple distance-based strategy used to disseminate information over a dense urban vehicular network. Upon receiving a data packet, the vehicle holds it for a time interval that is proportional to the reciprocal degree of the distance to the transmitting vehicle. Thus, it will be preferable to use vehicles situated farther from the transmitting vehicle to disseminate the information. Vehicles that hear the transmission of a packet that is already scheduled, can then cancel their transmission to avoid the broadcast storm problem. This approach can effectively handle the broadcast storm. However, this technique may encounter two problems: (i) the delay to deliver messages can be high, since there is no guarantee of the existence of vehicles close to the communication radius (i.e. those that will transmit with the lowest delay), and (ii) the coverage can be low since the vehicles will cancel their transmission indiscriminately when they hear the retransmission of the same packet.

Schwartz et al. [19] describe the Simple and Robust Dissemination (SRD) protocol, which was designed to operate in both dense and sparse highway vehicular networks and is an improvement on DV-CAST. Like DV-CAST, it relies exclusively on local one-hop neighbor information and does not employ any special infrastructure. Its main improvement (when compared with DV-CAST), is the idea of employing the optimized slotted-1-persistence broadcast suppression technique. Through this scheme, vehicles have different priorities for rebroadcasting which depend on their moving direction. SRD avoids the broadcast storm problem in dense networks and deals with disconnected networks by relying on the store-carry-forward communication model. However, the use of this protocol is based on certain assumptions that may not be realistic, such as the notion that all vehicles have the same communication range and a predefined direction for data dissemination.

Villas et al. [20] propose the GEographical Data Dissemination for Alert Information (GEDDAI), which uses a zone of preference (sweet spot) to eliminate the broadcast storm problem. In GEDDAI, upon receiving a data packet, each vehicle computes the waiting time before continuing the dissemination process, on the basis of the sweet spot and its position. Thus, it will be preferable to use vehicles inside the sweet spot to enable the data dissemination to continue. Vehicles that hear the transmission of a packet that is already scheduled, cancel their transmission to avoid the broadcast storm problem. This approach can effectively handle the broadcast storm. However, the GEDDAI protocol does not address the intermittent network problem.

Our proposal is based on neural networks and seeks to detect and control the congestion of routes through communication between vehicles. Unlike some other methods, the use of neural networks enables new situations to be learned, and thus allows a better response to new events and conditions.

### 2.2 Classify and Manage Traffic System of the Roads

The congestion control mechanism is designed to identify/classify, predict, and reduce congestion; when it detects a traffic jam, it classifies it according to its degree of severity. The literature on VANET includes some simple approaches to employing congestion detection and monitoring mechanisms, such as the proposals for monitoring and detecting traffic conditions using V2V (Vehicle-to-vehicle) set out in [21] and [22]. In this work, beacon messages are transmitted, which have low overhead and are used to notify the presence of a vehicle to its neighbors. Other approaches, such as ConProVa [23], only rely on V2I communication (Vehicle-to-Infrastructure), and implement a middleware to help in decision-making and the resolution of conflicts of interest arising from the current traffic conditions reported by vehicles. The reason for these conflicts is that vehicles are unable to validate the conditions detected on the road in a collaborative way. However, in practice, this approach has a drawback because it is limited to a single infrastructure.

*Pan et al.* [24] propose a centralized system to find out the vehicle geographic, speed and direction in real time to detect a traffic jam. Once detected, the vehicles that are approaching the traffic jam can be re-routed by applying three different algorithms. The first is *Dynamic Shortest Path* (DSP) which is the default in the system. It routes traffic to the shortest path at the lowest travel time. The advantage of this algorithm is its simplicity and as a result, it has a reasonable computational cost, where E is the number of road segments and V is the number of intersections. On the other hand, it has one drawback which is that the congestion may move to another spot. The second algorithm is *Random k Shortest Paths* (RkSP). RkSP randomly chooses a route from among the *k* shortest path routes. The purpose of this algorithm is to avoid switching congestion from one spot to another by balancing the re-routed traffic between several paths. As a result, RkSP has a higher computational complexity, which increases linearly with k. *Entropy Balanced k Shortest Paths* (EBkSP) improves RkSP by taking account of the effect that each *k* route has on the future pattern of traffic density. The result shows a reduction of average travel time of 36% for DSP, 41% for RkSP and 45% for EBkSP in the evaluated scenarios. Although this is a significant improvement, this approach requires a central controller and a periodic collection of information on vehicles and road segments, which makes it impractical for large urban area.

Wei-Hsun Lee et al. [25] fitted RSUs on traffic lights to send broadcast messages to the nearby vehicles and inform them about countdown traffic lights and the related problem of queuing. When they receive the messages, vehicles can determine the best driving speed. Doolan and Muntean [26] introduce EcoTrec, a novel eco-friendly routing algorithm for vehicular traffic that relies on vehicle-to-vehicle (V2V) communication. EcoTrec considers the road conditions and existing volume of traffic so that it can improve fuel consumption and reduce gas emissions. As a result, vehicles can adjust their routes and control their fuel consumption while on their trip. EcoTrec uses these data as metrics to determine the weight of each edge value in a graph model, and then chooses the best routes and redirects the vehicles. However, if an accident occurs, EcoTrec does not have any mechanism to prevent a congestion.

Our proposal allows different vehicle traffic congestion levels to be classified without an overload of control information in the network. Collisions can be reduced through this control information and by the greater flow of information exchanged. The proposed classification mechanism is intended to be a tool to inform drivers of the conditions on streets, so that drivers can make decisions wisely as to whether to change their route or not.

## 3 INCIDEnT—INtelligent protocol of CongestIon DETection

In this section describes the INCIDEnT protocol, first of all, we present an overview of our approach, follow by its components, (i) artificial neural network, (ii) detection and classification of congestion levels, (iii) data dissemination, (iv) exchanging Route.

### 3.1 Overview of our approach

**Fig 1:**
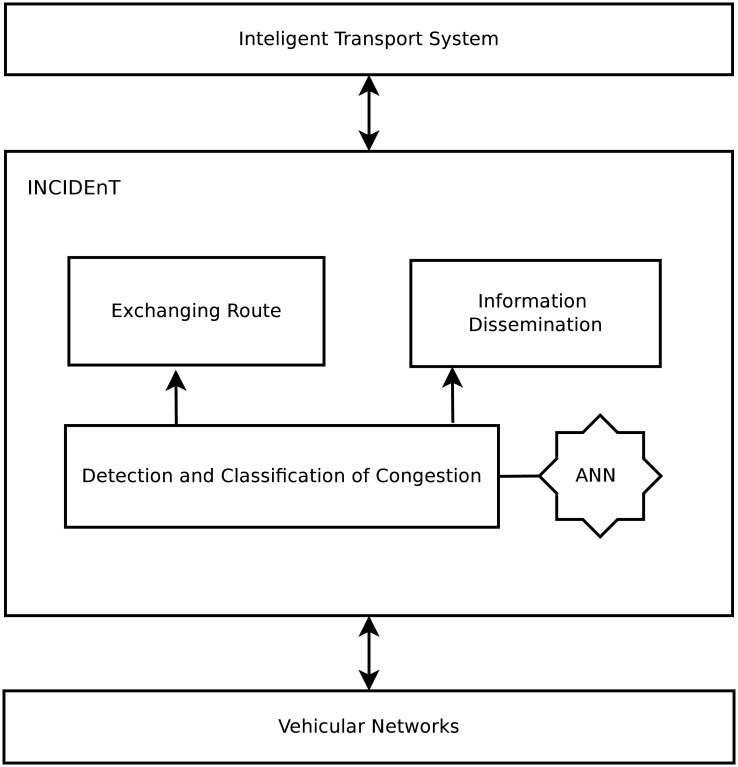
Main Components of INCIDEnT.

This section outlines the proposed solution for the data dissemination, detection, and control of congested roads in any environment, called INtelligent protocol of CongestIon DETection—INCIDEnT. The main purpose of INCIDEnT is to reduce congestion and improve traffic flow through a collaborative information transfer system between vehicles to reduce the average travel time, and also to lower fuel consumption and CO emissions. INCIDEnT is based on an Artificial Neural Network (ANN) that is designed to detect and classify the levels of congestion on roads, as well as suggest new routes for drivers to avoid congested roads. The system is divided into four main components: Artificial Neural Network, Detection, and Classification of Congestion, Information Dissemination, and Exchanging Routes as can be seen in Fig 1. In the following, we will describe each component of INCIDEnT.

### 3.2 Artificial Neural Network component

ANN is designed to detect and classify the levels of congestion on roads as well as to suggest new routes for drivers so that they can avoid them. The decision to use ANN, was based on its generalizing and self-learning abilities for detecting congestion levels, as well as its ability to adapt to new situations. Our proposed ANN relies on the vehicle speed and density of neighboring vehicles as input parameters. To estimate the speed and density, we based on [14, 27]. The speed value (*V*) is obtained through the GPS, which provides the speed, location, and direction of the vehicle. The density of neighboring vehicles on the track can be obtained from the periodical beacon messages by counting the number of received messages. As can be seen, each vehicle estimates the number of neighboring vehicles (*N*) in a time interval (*δT*). Hence, each vehicle is able to calculate the vehicle density (*ρ*) within a range of communication of one hop. In other words, the vehicle computes the number of adjacent vehicles within the same coverage area (*r*). The neighborhood density *ρ* is estimated by the relation $\rho =\frac{N}{r}$.

**Fig 2:**
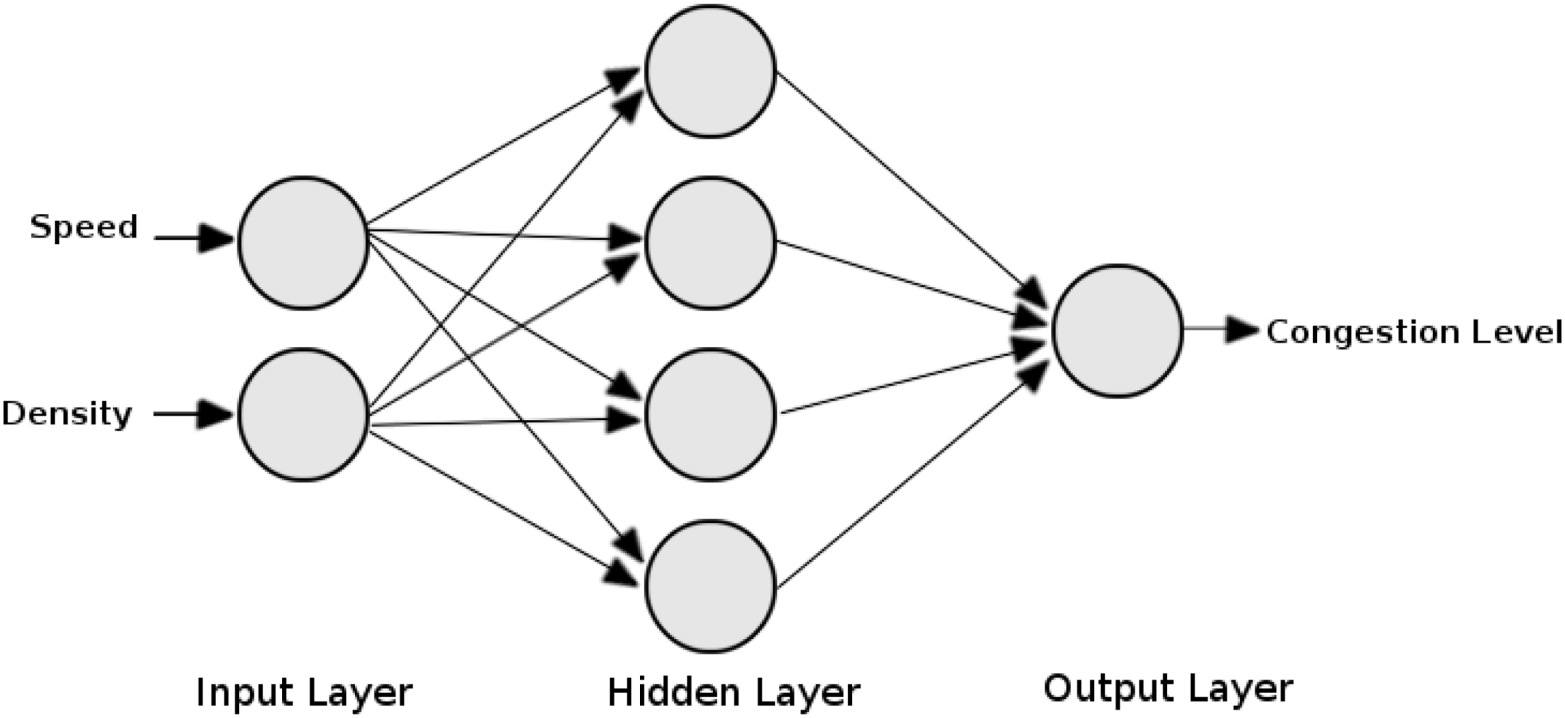
ANN topology employed in our proposal for detecting and classifying congestion levels.

The type of ANN employed in our model is the Multi-Layer Perceptron, which is designed to find out the congestion levels in urban environments. The choice of ANN that used in our model is justified by: (i) does not need, every dissemination of data, changing the network topology [28]; (ii) generalize congestion detection in urban and highway environments; and (iii) has promising results in other areas of research [29, 30]. ANN was configured with the following topology/features (Fig 2): (i) two neurons at the input layer, representing the vehicle speed and density of neighboring vehicles; (ii) a hidden layer with four neurons, representing the learning ability, which can classify the congestion levels, it should be noted that four neurons were sufficient to obtain a high learning rate, as presented in [31]; and (iii) an output layer neuron representing the classification of the level of congestion on the roads. The data of the output layer are standardized, and we utilized the hyperbolic tangent (*tanh*()) as the activation function. The hyperbolic tangent is a hyperbolic function which is obtained from the ratio of the hyperbolic sine and hyperbolic cosine. This is similar to the trigonometric relationship of the tangent. Moreover, we used the back-propagation algorithm to train the network that consists of two stages: (i) forward, in which data are propagated from the input layer to the output layer to obtain the output supplied from the network; and (ii) backward, in which uses the output supplied from the previous stages subtracted from the desired output to adjust the network weights. It should be noted that three levels of congestion were defined in our model, which are explained in Table 1

**Table 1:** Levels of Congestion.

Levels	Speedy(km/h)	Density(vehicles)	Output
**Free**	45–82	25–50	< 0.3
**Moderate**	16–40	51–65	> 0.3 and <0.7
**Congested**	< 40	> 65	> 0.7

It should be emphasized that following the training stage, we employed the obtained weights to classify the traffic congestion of the streets and roads correctly. We have chosen three output levels because we seek greater flexibility in decision time, that is, the system does not delay to converge, giving a faster classification, moreover, the mechanism will make a more assertive decision because there are not a number of states to evaluate it.

### 3.3 Detection and classification of congestion levels component

The detection and classification of the level of congestion are performed by all vehicles and carried out periodically every 2 seconds. This ensures, that we can provide instant information about the current state of the road for vehicles. Furthermore, we are able to prevent any precipitation in the computing classification and allow the classifier to establish a correct convergence of the level of the jam. This mechanism is shown in Algorithm 1, which carries out the detection and classification of congestion levels locally. This involves calculating the output of each neuron network,by using the weights obtained by the network training as well as the activation function. This can be seen in lines 5 to 9, after the classification has been completed (i.e. lines 10-19).

**Algorithm 1:** Classifies the level of congestion locally

1: *p*_1_ ← *getInputWeight*()

2: *p*_2_ ← *getIntermediateWeight*()

3: *v* ← *getSpeedy*()

4: *d* ← *getDensity*()

5: **for** each neuron **do**

6:  *Sum* ← *v* * *p*_1_[*i*] + *d* * *p*_1_[*i*+1]

7:  *OutputInter* ← *tanh(Sum)*

8:  *SumOutput* ← *SumOutput* + (*p*_2_[*i*] * *OutputInter*)

9: **end for**

10: *Out* ← *tanh(SumOutput)*

11: **if** (*Out* < = 0.3) **then**

12:  **return** *Free*

13: **end if**

14: **if** (0.3 < *Out* < 0.7) **then**

15:  **return** *Moderate*

16: **end if**

17: **if** (*Out* > = 0.7) **then**

18:  **return** *Congested*

19: **end if**

Our model also uses control messages (i.e. beacons) to make the information about the state of the roads available to all the vehicles. Each message consists of the location, speed, and direction, as well as the status of the particular road segment in which the vehicle is currently located. In our proposal, we compute the level of congestion of the current location of the vehicle, as well as the level of congestion of the route that the vehicle is approaching. The beacon exchange is required to: (1) disseminate the information about the level of congestion and routes (and this might cause a large overhead on the network); (2) anticipate other points of congestion that the driver can browse while driving (and this means displaying congestion information of the current situation and other segments as well the current one).

The algorithm 2 illustrates the operation for sending the beacon across the network. Each vehicle sends a beacon containing its speed, location, direction and the current state of its road segment, which is classified by our ANN. The vehicles also forward information about other segments, which form a part of the route for each vehicle and its particular location. If the vehicle has no knowledge of the congestion levels pertaining to other segments (but only the current one), these two values will be given as null. However, if the vehicle has this knowledge, it is able to establish the location and the level of congestion of the other segments that form a part of the route to the vehicle’s destination.

**Algorithm 2:** Method of Sending Beacon

 *beacon_mes* ← *Createmessage*()

2: *beacon_mes.speedy* ← *getSpeedy*

 *beacon_mes.local1* ← *getLocation*

4: *beacon_mes.direction* ← *getDirection*

 *beacon_mes.status1* ← *getLocalStatus(getLocation)*

6: **if** (*RecoverStatus* = = *Null*) **then**

  *beacon_mes.local2* ← *Null*

8:  *beacon_mes.status2* ← *Null*

 **else**

10: *beacon_mes.local2* ← *Status[1].local*

  *beacon_mes.status2* ← *Status[1].status*

12: **end if**

  *sendBeacon(beacon_message)*

Our mechanism is also used for a data dissemination scheme, should the vehicle have any information that needs to be disseminated. This information might be an event taking place on the road/street or some data relevant to the vehicle or even data from the Internet.

### 3.4 Data dissemination component

When there is a need to disseminate information, the vehicle creates a message that will be propagated to all the vehicles that are within the Area of Interest (AoI), which is the geographic location where the information has to be sent. Upon receipt of the packet, each vehicle checks if it is within the AoI or not by analyzing its geographical position. If not, the vehicle discards the received message. If it is within the AoI, then the vehicle checks the level of congestion and schedules the retransmission of the message, which is again based on the congestion level.

Since the level of congestion will slow the relay, the vehicle will check for duplication in the network, and in the event of there being a duplication, the algorithm aborts the transmission and deletes the message. In this way, it reduces the problem of broadcast storms. The mechanism for the dissemination of information based on the congestion level is shown in Algorithm 3.

**Algorithm 3:** Calculates the wait time to schedule the transmission based on the congestion level

1: $\text{distToSender}\leftarrow \sqrt{{\left(s_{x}-r_{x}\right)}^{2}+{\left(s_{y}-r_{y}\right)}^{2}}$

2: $\text{defaultDelay}\leftarrow 0.01\times (\frac{\text{distToSender}}{\text{communicationRadius}})$

3: **if** “Level == Free” **then**

4:  *delay* ← *defaultDelay* + random(0,0.02)

5: **end if**

6: **if** “Level == Moderate” **then**

7:  *delay* ← *defaultDelay* +random(0.03,0.06)

8: **end if**

9: **if** “Level == Congested” **then**

10:  *delay* ← *defaultDelay* + random(0.07,0.1)

11: **end if**

Whenever possible, the network fragmentation was detected by a vehicle, and this fragmentation is indicated by the absence of beacons. The vehicle stores the received information and uses its mobility to convey it to different parts of the area of interest. Furthermore, the vehicle can determine whether another vehicle has already received the information or not. For this reason, the vehicles are the store-carry-forward communication model, based on beacons sent by vehicles. The beacons are also used as an implicit recognition mechanism for confirming receipt of the information. Algorithm 4 shows the store-carry-forward mechanism that will store the information if the vehicle detects a fragmented network. The efPropagate is used to control the transmissions of a given vehicle, i.e., if it is below an acceptable threshold, the node broadcasts the information, otherwise it does not. Periodically, the vehicle calculates the efficiency of its message delivery.

Efficiency=Transmission/Beacons (1)

**Algorithm 4:** Mechanism of store-carry-forward

1: **while** “has not received beacons” **do**

2:  wait

3: **end while**

4: **if** (*efPropagate* = **true**)&&(isInsideAOI(beacon.sender)) **then**

5:  **if** (*ttlMesssage* ≠ 0) **and** (*hops* < *threshold*) **then**

6:   sendMessage()

7:  **else**

8:   delMessage()

9:  **end if**

10: **end if**

### 3.5 Exchanging Route component

We also rely on beacon messages for the route exchange mechanism. Algorithm 5 illustrates the set of actions performed when a beacon is received. When a vehicle receives a beacon, it checks if the segments belong to its path (line 2). If so, it determines their current status (line 4). If congestion is identified in its path, the vehicle starts to search for a new route and stores that information (lines 5-9). If moderate traffic or only a traffic-free roads are in its path, the vehicle simply stores that information (lines 10-17). If it receives a message with segments that are already in its records, the vehicle checks whether the state has changed from the last reading. The vehicle performs the same procedure for the other segment as we see in algortimo 5 lines 18—32

Through the above-mentioned strategies, our proposal achieves a reduction of CO emissions and lower fuel consumption, while the conductors reduce the travel time. A performance evaluation is conducted in the next section to validate our proposal.

## 4 Performance Analysis

This section describes the scenario and provides a performance assessment of the proposed solution by means of simulated experiments using the OMNeT++ 4.6 [32] network simulator, an event-based network simulator. We also use the Simulator for Urban MObility 0.21.0 (SUMO) [33] to build the scenarios and the vehicle mobility model. Furthermore, the experiments rely on the network framework Veins 4.2 [34], a well-known tool in the research community that implements the IEEE 802.11p protocol stack, signal attenuation caused by obstacles and other features made use of in our analysis.

**Algorithm 5:** Method of Receiving Beacon

 *beacon_mes* ← *DecapsulatePackage*()

2: *lack_route* ← *getLackroute*(*Currentlocation*)

 **if** ((*beacon_mes.local1* = = *lack_route*)) **then**

4:  **if** ((*beacon_mes.status1* = = *Congestion*) **then**

   *altRoutes* ← *Route().exclude(beacon_mes.local1*)

6:   *newRoute* ← *shortestPath(alterRoutes)*

   *changeRoute(newRoute)*

8:   *Status[firsts].local* ← *beacon_mes.local1*

   *Status[firsts].Status* ← *Congestion*

10:  **else**

   *Status[middle].local* ← *beacon_mes.local1*

12:  *Status[middle].Status* ← *beacon_mes.status1*

  **end if**

14: **else**

  *Status[lasts].local* ← *beacon_mes.local1*

16: *Status[lasts].Status* ← *beacon_mes.status1*

 **end if**

18: **if** ((*beacon_mes.local2* = = *lack_route*)) **then**

  **if** ((*beacon_mes.status2* = = *Congestion*) **then**

20:  *altRoutes* ← *Route().exclude(beacon_mes.local2*)

   *newRoute* ← *shortestPath(alterRoutes)*

22:  *changeRoute(newRoute)*

   *Status[firsts].local* ← *beacon_mes.local2*

24:  *Status[firsts].Status* ← *Congestion*

  **else**

26:  *Status[middle].local* ← *beacon_mes.local2*

   *Status[middle].Status* ← *beacon_mes.status2*

28: **end if**

 **else**

30:  *Status[lasts].local* ← *beacon_mes.local2*

  *Status[lasts].Status* ← *beacon_mes.status2*

32: **end if**

The EMIT model integrated into SUMO was employed to calculate CO emissions and fuel consumption. EMIT is a simple statistical model for estimating the instantaneous emissions and fuel consumption of vehicles based on speed and acceleration and is derived from the Handbook Emission Factors for Road Transport (HBEFA) [35] formula.

A realistic scenario was used for the simulations, taken from the OpenStreetMap [36] for both scenarios (i.e. urban (map and the area of interest are 1km) and highway scenarios (map and the area of interest are 30km)). The proposed INCIDEnT protocol was compared with four well-known solutions, GEDDAI [20], DBRS [17], SRD [19], and Linger [16] for evaluating the data dissemination. The second set of protocols, namely DSP [37] and RKSP [37] was adopted for evaluating the suggestion of router service on the intelligent transportation system.

In the following section, we describe the main metrics used to evaluate the INCIDEnT protocol, the simulation scenarios and the main results obtained.

### 4.1 Evaluated Metrics

The metrics used to compared the INCIDEnT protocol with GEDDAI [20], DBRS [17], SRD [19], and Linger [16] for evaluating the data dissemination. The second set of protocolos, namely DSP [37] and RKSP [37] are:

- *Coverage*: the relation between the number of vehicles within the area of interest when the data dissemination is achieved and the number of vehicles which received the data. Reliable data dissemination solutions are expected to achieve 100% coverage.
- *Total transmitted packets*: number of transmissions achieved during the data dissemination within the area of interest. A large number of packet transmissions is a strong indication that redundant messages are being disseminated, which may result in the broadcast storm problem.
- *Delay*: the average time required to achieve the data dissemination within the area of interest. A low delay is of particular interest to time-strict applications, such as the issuing of warning messages.
- *Message propagation distance*: the maximum average distance at which the data disseminated by the source vehicle was propagated within the area of interest. Reliable data dissemination solutions are expected to have a message propagation distance equal to the size of the area of interest.

An evaluation of when a system can be regarded as an intelligent transportation system must take into account the following criteria.

- Success rate: the rate of congestion correctly detected by the classification system;
- Trip time: the average travel time of all vehicles from point of origin to destination;
- CO emission: total amount of CO emissions produced during the journey of the vehicles;
- Fuel consumption: total amount of fuel consumption of vehicles moving from their point of origin to their destination.

### 4.2 Urban Environment

**Fig 3:**
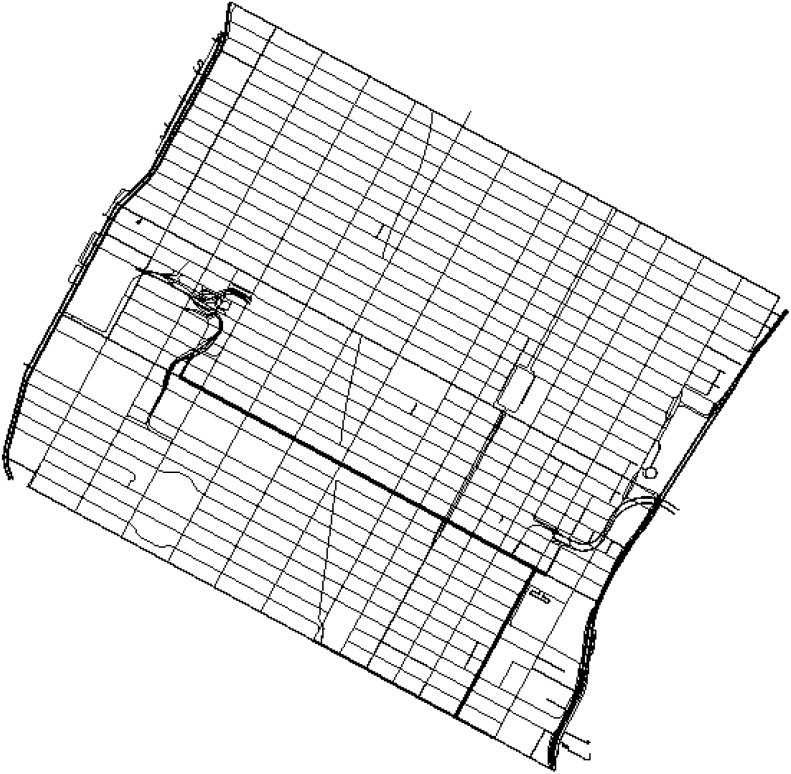
Manhattam neighborhood SUMO map.

We used the Manhattan map in New York, United States (see Fig 3). Vehicles travel at random along the street to simulate conditions close to real overtaking, and their speeds range from 30 to 60 kilometres per hour; the conditions of traffic are 1000, 2000, 3000, 4000 and 5000 vehicles/Km^2^. This large amount of traffic in the map causes congestion on the streets.

The simulation time is 7000 seconds, which is long enough to evaluate the proposed solution while taking account of the evaluated metrics. This also allows the simulator to remain in a stable condition for a significant amount of time during the simulation. In other words, this simulation time allows all the vehicles to effectively travel through the map. Vehicles use the IEEE 802.11p as the underlying network protocol.

Owing to the wide variation of vehicle density and to avoid overloading the network, we set the beacon shipping frequency to 2 Hz [38]; the update frequency of the propagation efficiency was also performed in every 1 s. Finally, in all the results obtained, each point in the graphs represents the average of 40 repetitions with a confidence interval of 95%. Table 2 provides a summary of the configuration parameters that were employed to execute the simulations.

**Table 2:** Simulation parameters.

Parameters	Value
Transmission power	[0.98]mW
Transmission range	[200]m
Bit rate	[18]Mbit/s
Beacons rate	[2]Hz
Number of runs	40
Confidence interval	95%

#### 4.2.1 Results of the Data dissemination

**Fig 4:**
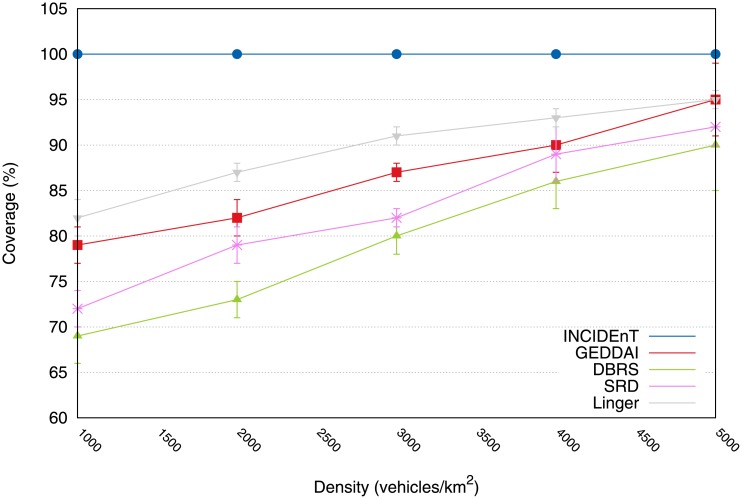
Coverage according to density. Our proposal shows that it achieved 100% of coverage for all densities.

The coverage metric was used to evaluate the efficiency of the proposed protocol for the data dissemination in networks with different traffic conditions. Fig 4 shows that the proposed protocol achieves the best performance for all traffic conditions when compared with the SRD, DBRS, GEDDAI and Linger protocols. These results can be explained by the fact that the proposed protocol allows vehicles within the AoI to store and carry the data packet around the network until the vehicle reaches a new one that has not received the message yet. When this occurs, the informed vehicle forwards the data to the uninformed vehicle.

On the other hand, the solutions available in the literature halt the dissemination process (for lower densities) at the first network disconnection, and this results in a poor delivery rate. The reason for this is that they are unable to rely on the store-carry-forward communication model. Hence, we believe that our proposed protocol is well suited for application scenarios that require reliable data delivery.

**Fig 5:**
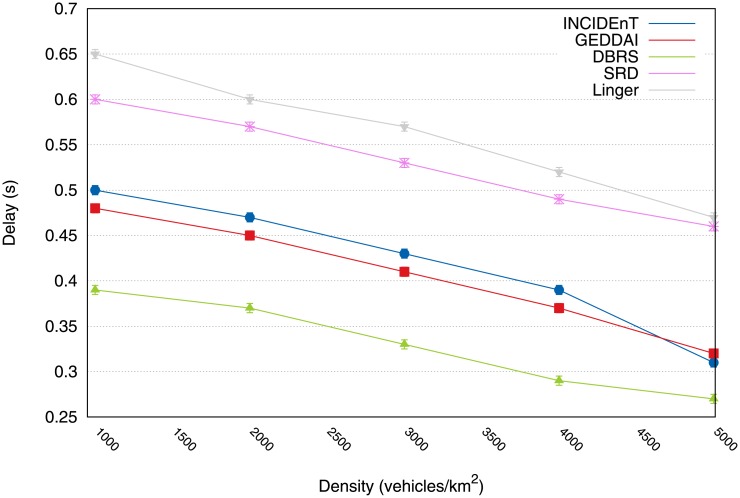
Results of average delay for delivering packets to intended recipients.

Fig 5 shows the average incurred delay in delivering data packets to the intended recipients. These results show that although the delay is slightly greater delay than with the Linger protocol, our protocol is a more suitable solution whenever reliable data delivery is required within strict time limits (see Fig 4).

**Fig 6:**
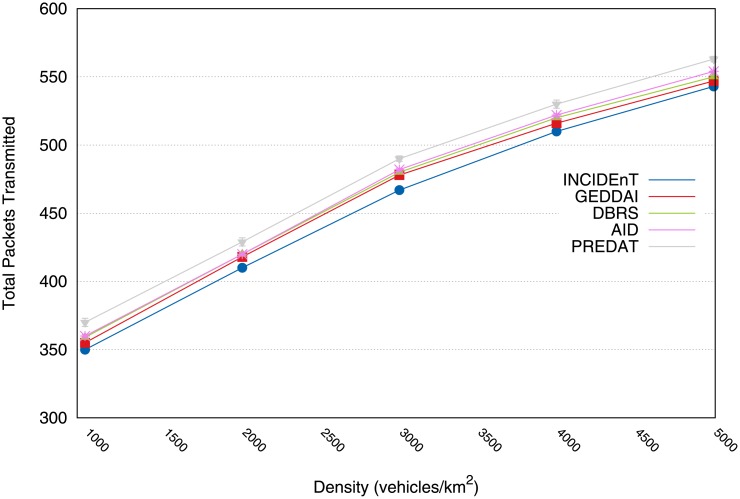
Transmitted packets by vehicles during the dissemination process.

Fig 6 shows the total number of data packets transmitted by all the vehicles during the data dissemination. The Linger protocol incurred a higher overhead than the others. Our protocol disseminates about 18% fewer packets than that of Linger. This is due to its broadcast suppression mechanism which is what we built on. Moreover, our protocol disseminates approximately the same amount as SRD, DBRS and GEDDAI do. In fact, for higher densities, our solution produces a slightly lower overhead than these solutions. That said, we argue that our modeled protocol does not waste bandwidth by including unnecessary rebroadcasts.

**Fig 7:**
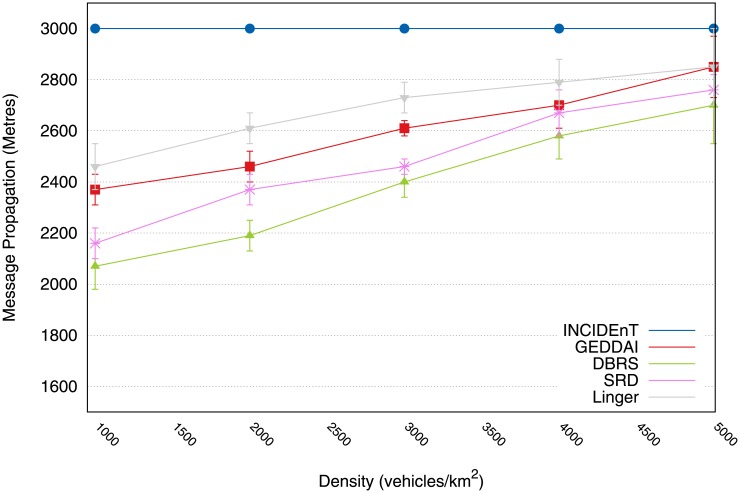
Performance of message propagation in meters achieved by each protocol.

Finally, Fig 7 shows the distance at which each message is capable of being transmitted. The message propagation distance is worth evaluating, as it measures the protocol’s efficiency during the data dissemination process. This is because most applications require data to be disseminated in the largest possible area of interest.

Fig 7 shows that our protocol provides a better performance than the other protocols. It should be noted that during the data dissemination in the area of interest, Linger, SRD, AID and GEDDAI are able to propagate data to around 85% of the area of interest, while our version/protocol is able to propagate data to more than 99% of the area of interest (see Fig 7). This is because our protocol makes use of a store-carry-forward mechanism to deal with network partitions.

#### 4.2.2 Results of Suggested Route

**Fig 8:**
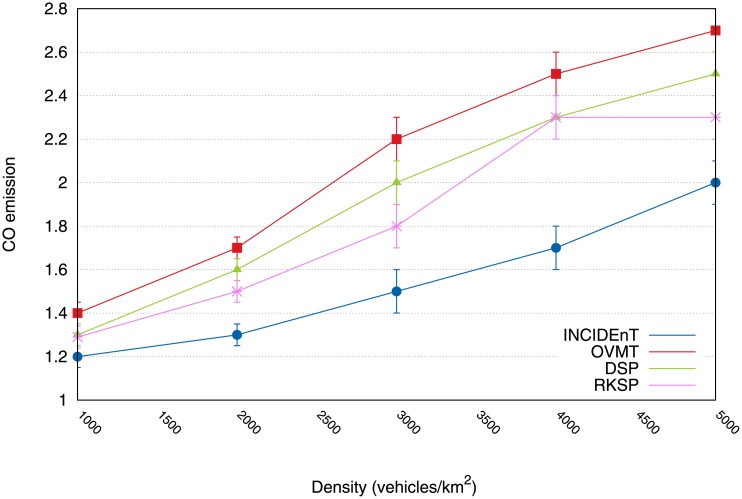
CO emission reduction achieved by each protocol.

There will now be an evaluation of how we employ intelligent transport systems as a means of reducing CO emissions, fuel consumption, and travel time in urban scenarios. Fig 8 shows that our protocol enabled us to reduce CO emissions, in contrast with the mechanism that has no route changes. When there were 5000 vehicles/km^2^ and OVMT, INCIDEnT obtained a reduction of approximately 35% and when compared with the protocol that is closest to our proposal, it obtained a reduction of 15%.

With fewer vehicles, there was a smaller reduction, as for example 10% with 1000 vehicles/km^2^. In all of the evaluated scenarios, there was a significant reduction in the emission of CO, depending on the nature of each protocol. It should be pointed out that traffic congestions increase the amount of CO emissions and by avoiding these areas, drivers can help to reduce these emissions.

**Fig 9:**
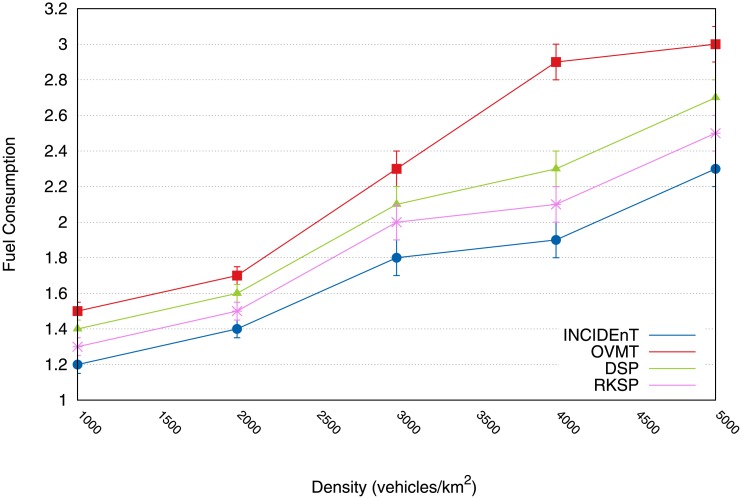
Fuel consumption consumed by vehicles relying on each protocol.

Fig 9 shows the results computed for fuel consumption. Changing the route scenario resulted in lower fuel consumption when the route change was set to disabled mode. When an analysis was conducted of 1000 vehicles/km^2^, our protocol obtained a reduction of 8% in fuel consumption. In the case of 5000 vehicles/km^2^, our model achieved a 23% greater reduction than OVMT and 10% greater than DSR and RKSP. This reduction was possible because a new route was taken after the drivers had been given information regarding the location of the event which caused the congestion.

**Fig 10:**
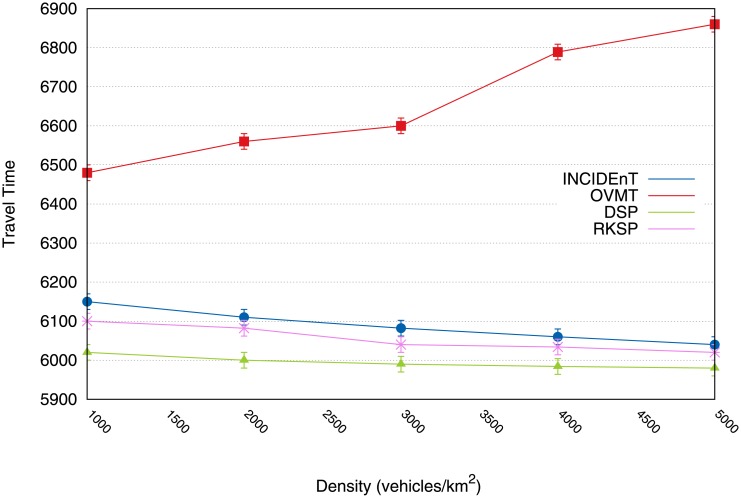
Elapsed trip time with each disseminating protocol.

With regard to the trip time (Fig 10), our protocol obtained a reduction of 9%. In the case of 5000 vehicles/km^2^, our solution achieved a 26% greater reduction than OVMT, and achieved a 2% greater reduction than DSR and RKSP. Bearing this in mind, we demonstrated that the proposed algorithm achieved significant results in reducing CO emissions, fuel consumption, and trip time. Moreover, our proposal achieved a good level of accuracy in its classification of congestion levels and allowed vehicles to schedule the messaging within an acceptable time.

### 4.3 Experiments with the highway environment

The following highway location was adopted for this scenario: 30km West of SP-065, Dom Pedro I freeway. This road in the State of Sao Paulo connects the Anhanguera and Dutra highways and serves the cities of Campinas, Atibaia, and Sao Jose dos Campos. Vehicles can move in both directions along the highway. In addition, vehicles are deployed at opposite ends of the road at a constant density (1000, 2000, 3000, 4000 and 5000 vehicles/hour).

Three types of vehicle were employed to form the most realistic simulation. The first consists of vehicles capable of reaching a top speed of 120km/h (the highway speed limit) and each vehicle is four meters long. The second consists of vehicles capable of reaching a top speed of 90km/h and are 14 meters long. Finally, the third type comprises vehicles of 18 meters, which are capable of reaching a top speed of 90km/h. This scenario describes a network made up of passenger cars, buses and trucks. The network consists of 50% cars, 25% buses and 25% trucks.

When the simulation reaches a stable state and is able to generate congestion on a freeway, an accident takes place on the highway and the vehicles involved produce a single data packet. The data packet corresponds to information of an event on the road (i.e. an accident), which is being disseminated through a multi-hop communication to notify drivers approaching the accident site. The aim is to disseminate the accident information within a reasonable period of time so as to ensure that the information reaches the widest possible range of vehicles approaching the accident area.

#### 4.3.1 Results of the data dissemination in a highway environment

**Fig 11:**
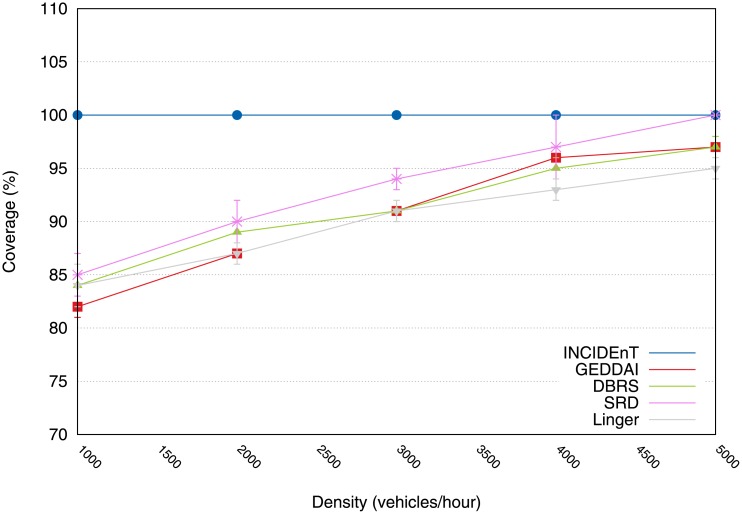
Snapshot of message transmission coverage using several protocols.

Fig 11 shows the coverage result for all the protocols in varying traffic conditions. As can be observed, our protocol is the best performing protocol in all traffic conditions. The solution delivers the message to about 100% of the intended recipients, while the other protocols deliver to (at most) 90% of the vehicles. This is due to the preemptive mechanism that prevents data losses, if there is a network partition. Unlike the other protocols, where messages are simply received by a vehicle and then broadcast and discarded, the INCIDEnT protocol allows data to be stored until the vehicle receives a new beacon, if there are no vehicles within the communication range. This result is evidence that our model is a suitable solution for scenarios that require reliable data delivery.

**Fig 12:**
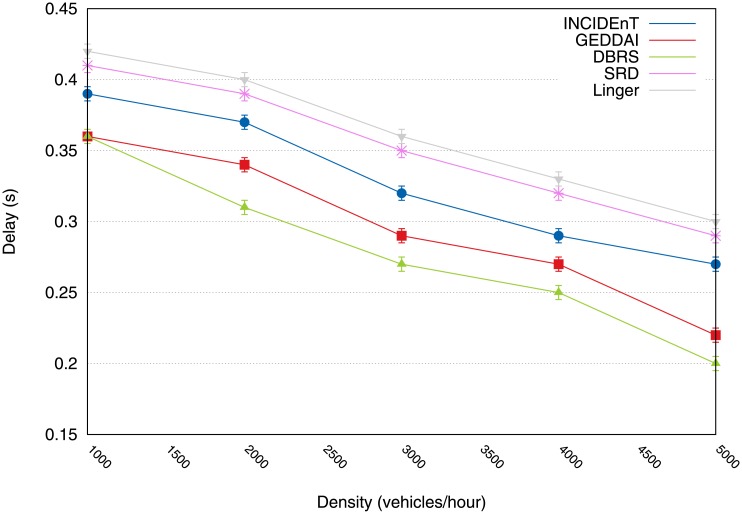
Average delay to deliver packets into intended recipients.

Fig 12 shows the average delay that occurs when delivering the data packets to intended recipients. This result shows that despite a slightly longer delay than that experienced by Linger and SRD, the proposed protocol is the only acceptable solution for applications that require reliable data delivery within a strict time limit (see Fig 11).

**Fig 13:**
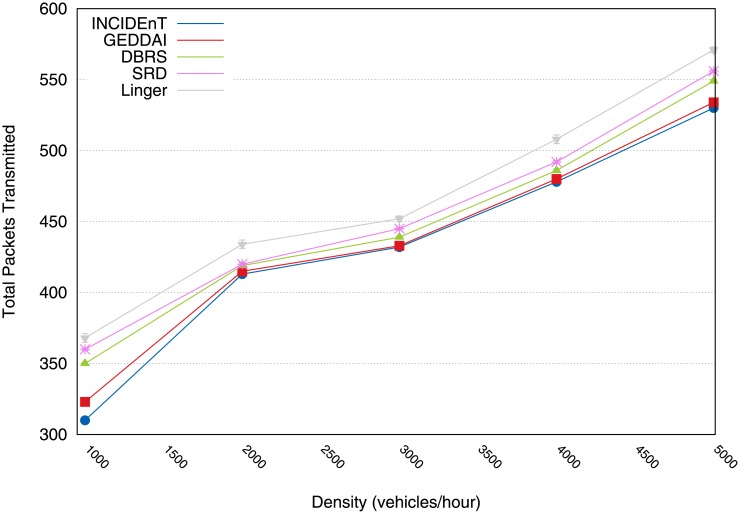
Total number of transmitted packets during the dissemination process.

Fig 13 shows the total number of data packets transmitted by all the vehicles during the dissemination process for different traffic conditions. The proposed protocol retransmits many of the stored messages when a vehicle realizes that there is a network partition and encounters a new vehicle able to continue the dissemination process. Our protocol has the same transmission rate as that of GEDDAI, although the proposed protocol increases the coverage of transmission (see Fig 11). This result suggests that the proposed protocol can deliver data to the intended recipients reliably, within a strict time limit in varying traffic situations s without incurring a high overhead in the network.

**Fig 14:**
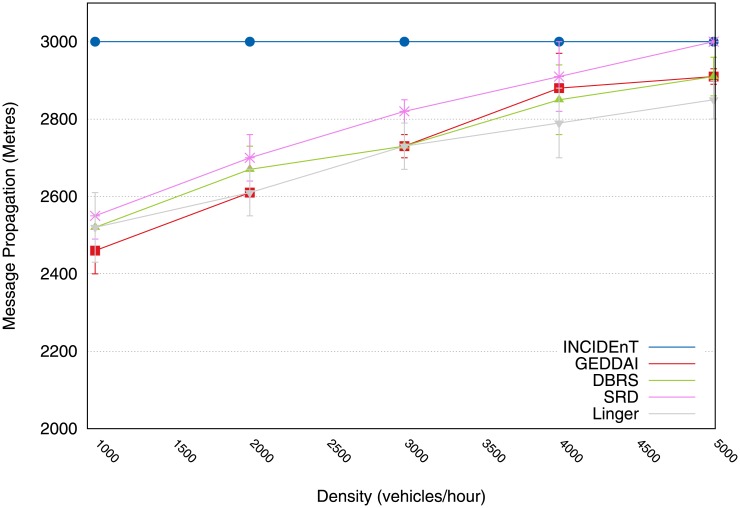
Maximum distance of message propagation in meters issued by vehicles within the AoI.

Finally, Fig 14 shows the average maximum distance in meters that each vehicle disseminated within the area of interest. It shows that our protocol achieved a better performance than the other protocols. It was observed that during the data dissemination in the area of interest with a length of 3 km, the DBRS protocol is able to propagate data to 80% of the area of interest, SRD to only 90%, Linger only 77%, whereas our solution to more than 100% (see Fig 14). With a low traffic density, our modeled protocol performs better than Linger because of its store-and-forward mechanism, which prevents the data from being lost if there are network partitions.

#### 4.3.2 Results of Suggested Route

**Fig 15:**
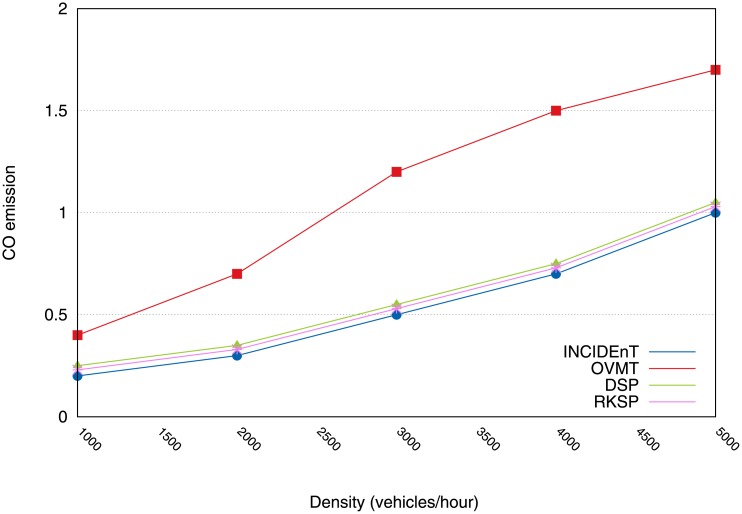
Reduction of CO emission using our protocol compared to other efforts.

We are now in a position to show the evaluation of the applicability of methods for intelligent transport systems which are aimed at reducing CO emissions, fuel consumption and travel time. Fig 15 shows that our protocol was able to reduce a higher degree of CO emissions than the other protocols. In the case of the spot with 5000 vehicles/hour, our solution obtained a reduction of approximately 58% of emissions. With fewer vehicles, we obtained a reduction of 50% in a traffic range of 1000 vehicles/hour.

Hence, for all the evaluated scenarios, we observed a significant reduction in the emission of CO because the vehicles disseminated information in an acceptable time to allow the vehicles to exchange their routes before reaching the congested areas. This was possible because there was a correct classification of the congestion level, including the number of vehicles close to the accident. This allowed a rapid classification, thus, it allowed the data to be transmitted within a reasonable time.

**Fig 16:**
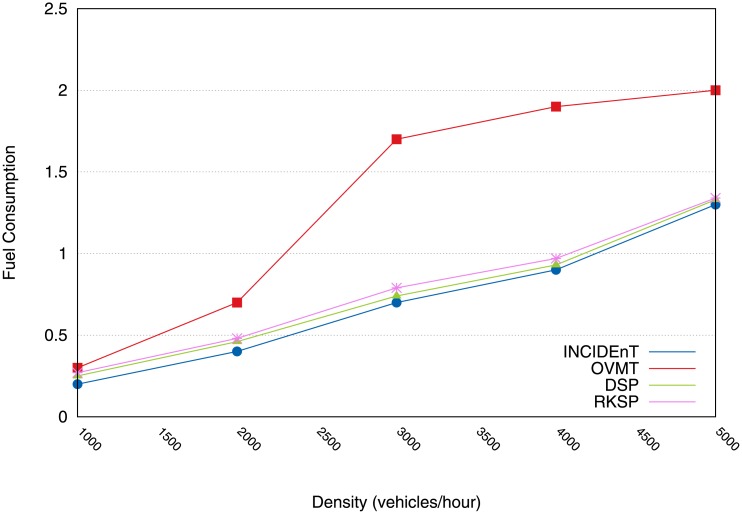
Fuel consumption reduction imposed by our solution & comparision to other protocols.

Fig 16 shows the fuel consumption. Our model requires less fuel consumption compared to OVMT. When the situation involving a rate of 1000 vehicles/hour was analyzed, the active mechanism achieved a 44% reduction in fuel consumption. In the case of 5000 vehicles/hour, our protocol obtained a reduction of 65% greater than OVMT and a reduction of 5% greater than DSK and RKSP. This reduction was made possible by the creation of new routes, as soon as the vehicle received the information about the congested areas.

Fig 16 shows the fuel consumption. Our model imposes less fuel consumption compared to the state when the vehicle engine is off. Analyzing the case of 1000 vehicles/hour, the active mechanism achieved a 44% reduction in fuel consumption. In the case of 5000 vehicles/hour, our protocol obtained a reduction of 65% compared to OVMT and a reduction of 5% compared to DSK and RKSP. This reduction was made possible thanks to the creation of new routes, as soon as the vehicle received the information of congested areas.

**Fig 17:**
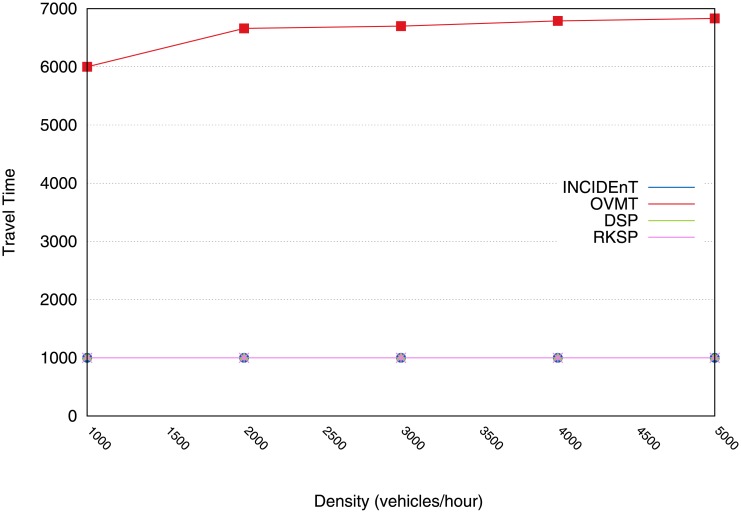
Elapsed trip time by vehicles using different protocols & including our proposed solution.

With regard to the trip time, our scheme kept the vehicle’s trip time to approximately 1000s. This was achieved by our dissemination protocol because few vehicles remained in the congested areas. However, OVMT resulted in an average of 6700s, as can be seen in Fig 17. Thus, this confirms that our proposed algorithm achieved good results in reducing CO emissions, fuel consumption and trip time. In addition, the mechanism obtained a good hit rate in terms of its ability to classify congestion levels and hence allow the vehicles to scale the messaging within an acceptable time.

### 4.4 Discussion of results

The obtained results show that in an urban scenario, our INCIDEnT protocol obtained a 100% coverage—a smaller amount of packets transmitted with a high rate of propagation. This was due to the detection mechanism and classification of congestion levels, which in turn performed the abstraction of network status and allotted the right amount of time for the transmission of information.

In our analysis of the applicability of the solution to an intelligent transport system, the INCIDEnT protocol achieved a low rate of fuel consumption, low CO emissions and a similar trip time to that of most of the other scenarios. This was due to exchange beacons among neighbors which allowed the abstraction of a significant amount of information; with this information, it was possible to perform a correct classification of congestion at a street level.

It should be emphasized that the highway scenario achieved roughly the same performance—a rate of coverage (100% of coverage). Although there was an average delay that was similar to that of other scenarios, the proposed protocol managed to transmit a smaller number of packets with a high rate of propagation. When the applicability of the solution to an intelligent transport system was analyzed, we observed a behavior that was close or similar to other scenarios.

On the basis of these results, it can be concluded that the protocol INCIDEnT experienced a similar behavior in both scenarios, although there may have been a minimal reduction when the protocol was used in road scenarios. This is because when there is less information due to the low number of vehicles, the protocol will achieve a significant performance in urban settings.

The proposed protocol relies on ANN in vehicles and hence only embodies the classification mechanism; the self-learning system has not yet been implemented in the vehicles. The reason for this is that the network topology is highly dynamic and the time duration that the vehicle has to process a message, transmit, and even complete its course, is relatively low. This hampers the implementation of a self-learning mechanism in the vehicle. It should be stressed that the convergence time of the network learning process can be high, which means that it can be suitably implemented in the RSUs. The computation for congestion classification of a given segment is relatively low, and does not have a direct impact on the performance of the INCIDEnT protocol. As was seen in the algorithm 1 the classification method only performs simple multiplication calculations and comparisons of instructions.

## 5 Final remarks

In this paper, we have proposed a solution for detecting and controlling congestion, which is based on inter-vehicle communications. The INCIDEnT is based on an artificial neural network (ANN) that is employed for detecting and classifying congestion levels. The simulation results show that the The INCIDEnT achieved a significant success rate in the level of congestion, by managing to reduce CO emissions, fuel consumption, and trip time. It should be stressed that we have used a realistic scenario for the simulations, which was borrowed from the OpenStreetMap. In future work, we intend to make new simulations to compared to classifications method.
